# Combined written and oral information prior to gastrointestinal endoscopy compared with oral information alone: a randomized trial

**DOI:** 10.1186/1471-230X-8-22

**Published:** 2008-06-03

**Authors:** Christian Felley, Thomas V Perneger, Isabelle Goulet, Catherine Rouillard, Nadereh Azar-Pey, Gian Dorta, Antoine Hadengue, Jean-Louis Frossard

**Affiliations:** 1 Division of Gastroenterology and Hepatology, University Hospitals, and University of Geneva, Geneva, Switzerland; 2 Division of Gastroenterology and Hepatology, University Hospital, and University of Lausanne, Lausanne, Switzerland; 3 Clinical Epidemiology Service, University Hospitals, Geneva, Switzerland

## Abstract

**Background:**

Little is known about how to most effectively deliver relevant information to patients scheduled for endoscopy.

**Methods:**

To assess the effects of combined written and oral information, compared with oral information alone on the quality of information before endoscopy and the level of anxiety. We designed a prospective study in two Swiss teaching hospitals which enrolled consecutive patients scheduled for endoscopy over a three-month period. Patients were randomized either to receiving, along with the appointment notice, an explanatory leaflet about the upcoming examination, or to oral information delivered by each patient's doctor. Evaluation of quality of information was rated on scales between 0 (none received) and 5 (excellent). The analysis of outcome variables was performed on the basis of intention to treat-analysis. Multivariate analysis of predictors of information scores was performed by linear regression analysis.

**Results:**

Of 718 eligible patients 577 (80%) returned their questionnaire. Patients who received written leaflets (N = 278) rated the quality of information they received higher than those informed verbally (N = 299), for all 8 quality-of-information items. Differences were significant regarding information about the risks of the procedure (3.24 versus 2.26, p < 0.001), how to prepare for the procedure (3.56 versus 3.23, p = 0.036), what to expect after the procedure (2.99 versus 2.59, p < 0.001), and the 8 quality-of-information items (3.35 versus 3.02, p = 0.002). The two groups reported similar levels of anxiety before procedure (p = 0.66), pain during procedure (p = 0.20), tolerability throughout the procedure (p = 0.76), problems after the procedure (p = 0.22), and overall rating of the procedure between poor and excellent (p = 0.82).

**Conclusion:**

Written information led to more favourable assessments of the quality of information and had no impact on patient anxiety nor on the overall assessment of the endoscopy. Because structured and comprehensive written information is perceived as beneficial by patients, gastroenterologists should clearly explain to their patients the risks, benefits and alternatives of endoscopic procedures. Trial registration: Current Controlled trial number: ISRCTN34382782.

## Background

Informed consent is a legal and an ethical requirement. Obtaining informed consent from patients who will undergo a medical procedure is of particular interest for the doctor, since failure to obtain informed consent increases the likelihood of malpractice lawsuits [1,2]. There is a broad variety of legal prescriptions regarding informed consent in different countries. In countries with strict legal situation such as Germany, all aspects of the information (content form, obtain informed consent, time, place and circumstances of endoscopy) are regulated in detail by legal authorities. In several European countries however, there is no legal requirement to obtain an informed consent before an endoscopic procedure, although many gastroenterologists use some kind of informed consent on a voluntary basis. Informing patients comprehensively is particularly important for procedures that are perceived as minor or safe, but that are known to carry a risk of complications. Digestive endoscopy falls in this category [3].

Guidelines suggest that patients should be informed about the following: 1) nature of the procedure, 2) procedure-related risks, 3) procedure benefits and 4) alternatives to the procedure [4]. In practice, however, patient information is often inadequate. In a survey of 157 USA hospitals, only 26% of informed consent forms adequately addressed all four points cited above [4]. This may be partly related to possible objections to extensive patient information. One objection is that the informed consent process may undermine trust between patient and doctors [5]. Another objection is that information may unnecessarily increase patient anxiety. Regarding this issue, limited evidence suggests that detailed information does not raise patient anxiety before general anesthesia [6], nor does it increase patient's fear before upper gastrointestinal endoscopy [7]. The risks and benefits of providing information to patients may depend on several factors such as: the medium (oral information, leaflet, videotape), the level of detail, the manner of presenting key evidence (e.g., relative or absolute risk), the possibility of asking questions, the cultural context, and of course individual patient preferences.

Given the uncertainty about the most effective way of informing patients, we conducted a randomized trial comparing the effects of written information leaflets, sent to patients by mail before digestive endoscopy, with the effects of the standard procedure used at our institutions, which consisted of verbal information delivered by the patient's doctor without specific guidance. We then assessed the impact of these information procedures on patients' perceptions of the quality of information they received and on their level of anxiety.

## Methods

### Study design and population

**Figure 1:**
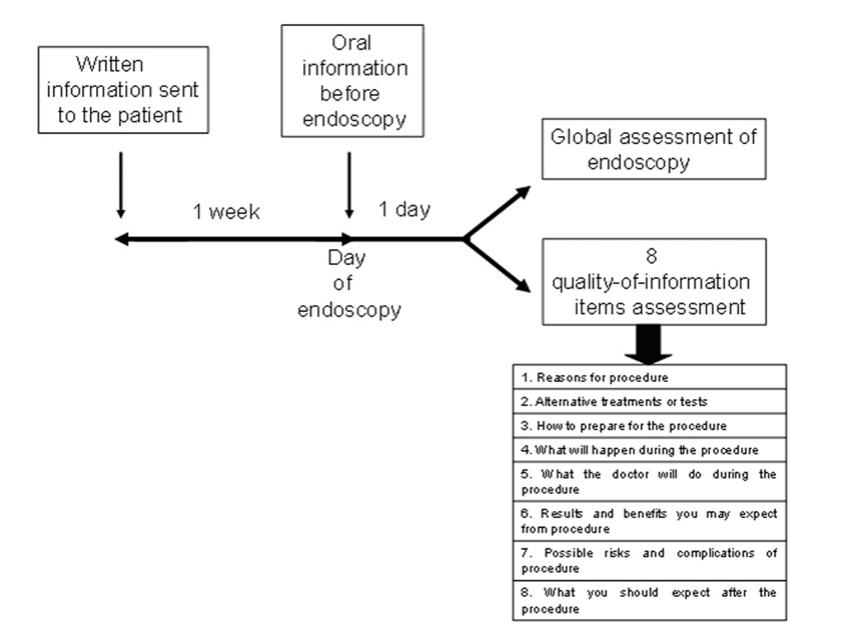
**Flow chart of the study design**: In the written and oral information group, patients received the written information one week before the planned endoscopy and oral information the day of the endoscopy. In the oral information group, patients only received oral routine non standardised information the day of the endoscopy. All patients were then asked to rate the endoscopy course (global assessment of endoscopy) and to response to the 8 quality-of-information items.

The study complied with the Declaration of Helsinki regarding investigation in humans and was approved by our institutional ethics committee (Commission centrale d'Ethique). This was an investigator-initiated study with no involvement of industry in the design, conduct, funding, or analysis of the results. We conducted a randomized controlled trial comparing the combination of written and oral information before endoscopy with only oral information given prior to endoscopy (Figure 1). In the written and oral information group, the written information regarding the planned procedure was sent to each patient at least one week before the scheduled date, whereas the oral information was given on the scheduled day of the procedure. In the oral group information, the information was given on the scheduled day of the procedure. The oral information consisted of routine non standardised oral information (but contained all information necessary to understand the benefits and risks of the endoscopy) provided by the prescribing physician before the procedure and by the gastroenterologist on the scheduled day of the procedure. Each and every patient could ask questions and have a discussion of oral consent on the day of the procedure. The written information contained all standard information that is regularly given to the patients: benefits and risks of the endoscopic procedure, treatment of endoscopy-related complications and possibility of receiving hypnotic drug during endoscopy and the related risks.

Inclusion criteria: were eligible all patients scheduled for an elective digestive endoscopy (upper gastrointestinal endoscopy or colonoscopy) on a three-month period allowing us to include at least 800 patients. Each patient should live in Switzerland, understand French language, and be able to fill in the study questionnaire. Both inpatients and outpatients were included. Exclusion criteria: age < 18 years, pregnancy, patients unable to give their own consent and patients that had already undergone prior endoscopy.

Randomisation between combined written and oral information and oral information alone was performed at the time the appointment was made at our institutions. Outpatients were referred by their own family physician for a procedure using a direct-access or open-access referral without prior evaluation in our hospitals. The appointment letter was sent to the patient with or without the written information leaflet usually within one week before endoscopy. Patient consent to participate was obtained only upon his arrival for the endoscopic procedure. Therefore, all patients signed an informed consent form upon their arrival.

### Study variables and data collection

The main outcome measures were the evaluation by the patient of various aspects of information received about the procedure that we defined as 8 quality-of-information items (Figure 1): the reasons for the procedure, alternative treatments or tests, how to prepare for the procedure, what the doctor will do during the procedure, results and benefits to be expected from the procedure, possible risks and complications. Possible response were no information given (scored as 0), poor (1), fair (2), good (3), very good (4), and excellent (5).

In addition, patients rated their anxiety at the time of the procedure (between none and strong), how tolerable the procedure was (between very easy and very hard), their pain during the procedure (between none and strong), whether any health problems occurred as a result of the procedure (none, minor, moderate or severe), as well as the procedure as a whole (between poor and excellent). Patients were also invited to rate any written information they received, in terms of usefulness (between very useful and totally useless), clarity (between very clear and very difficult to understand), and whether the information made them feel anxious or reassured. The latter items were relevant only for patients in the written information arm. Finally, we asked the nurse assisting the gastroenterologist to subjectively rate the course of the endoscopy (well-tolerated or not well-tolerated).

Other confounding variables included patient age, sex, French mother-tongue, level of education, type of procedure (upper gastrointestinal endoscopy or colonoscopy), duration of procedure, whether the patient was premedicated, whether a complementary intervention was performed (biopsy, excision, dilation), whether it was a first endoscopy, outpatient versus inpatient status, and selected comorbidities (health disease, lung disease, obesity, blood pressure treatment, and beta-blocker treatment).

All patients who were willing to participate in the study left the endoscopy unit with a questionnaire to be filled within 24 hours and sent back by mail in a prepaid envelope.

### Statistical analysis

First, we compared the baseline characteristics of the two study groups. The analysis of outcome variables was performed on the basis of intention to treat-analysis. We used primarily cross-tabulation of intervention by outcome variable (for ordinal variables), and tested linear trends by chi-square tests with 1 degree of freedom. For conciseness' sake, we showed means and medians of information ratings and tested them by means of Mann-Whitney tests. Multivariate analysis of predictors of mean information scores was performed by linear regression, using a stepwise procedure, with P < 0.05 as a criterion for inclusion and P > 0.05 as a criterion for exclusion of each covariate. The modelling was conducted under the analyst's control, not with an automated algorithm. P values < 0.05 were interpreted as statistically significant.

## Results

### Recruitment

In Lausanne, 500 patients were provisionally randomized, and 412 in Geneva over a three-month period. Of these, 66 (7.2%) patients were later found to be ineligible due to poor health, language barriers, or being enrolled in another study, 27 (3.0%) were withdrawn for technical problems (endoscopy performed before scheduled date, patient missed by personnel, lost record), and 101 (11.1%) cancelled their examination. More patients in the written information group cancelled their procedure or did not show up, as compared with patients who received oral information (Table 1). Of the 718 eligible patients, 27 (3.8%) refused to participate, 114 (15.9%) failed to send back their questionnaire, and 577 (80.4%) completed all study procedures. Participation rates were similar in the two groups.

**Table 1:** Characteristics of patients enrolled in randomised trial of written plus oral information versus oral information before digestive endoscopy, Lausanne and Geneva, 2000.

	Written and oral information	Oral information
Randomised initially, N	454	458
Not eligible, N (%)	37 (8.1)	29 (6.3)
Technical enrolment problem, N (%)	18 (4.0)	9 (2.0)
Cancelled procedure, N (%)	60 (13.2)*	41 (9.0)
Eligible for survey, N	339	379
Participated, N (%)	278 (82.0)	299 (78.9)
Respondents, N	278	299
Women, N (%)	135 (48.6)	139 (46.5)
Age, mean (SD)	57.0 (16.9)	58.7 (16.2)
Native French speaker, N (%)	193 (69.4)	190 (63.5)
Education beyond high school, N (%)	100 (36.5)	97 (32.8)
Gastroscopy (vs colonoscopy or both), N (%):	148 (53.2)	183 (61.2)
Duration of procedure, minutes, mean (SD)	30.8 (24.6)	27.3 (22.4)
Premedication, N (%)	202 (72.7)	205 (68.6)
Biopsy, polyp excision, dilation, N (%)	212 (76.3)	213 (71.2)
First endoscopy, N (%)	134 (48.2)	139 (46.5)
Hospitalised (vs outpatient), N (%)	100 (36.6)	120 (41.0)
Heart disease, N (%)	63 (22.7)	73 (24.4)
Lung disease, N (%)	22 (7.9)	18 (6.0)
Obesity, N (%)	31 (11.2)	22 (7.4)
Blood pressure treatment, N (%)	73 (26.3)	75 (25.1)
Beta-blocker treatment, N (%)	26 (9.4)	20 (6.7)

* P = 0.045, Fisher exact test

### Baseline comparisons

Among participants in the patient survey, the groups were similar in terms of socio-demographic variables, type and duration of the procedure, and prevalence of comorbid conditions and treatments and type of endoscopy (Table 1).

### Patient global assessment of endoscopy

Having received written information before the procedure did not change the patients' level of anxiety, the tolerance of the procedure, the pain experienced and the global evaluation of the procedure compared to oral information (Table 2). Furthermore, endoscopy nurses rated the procedure as having gone well for 221 (79.5%) patients who received written information, versus 239 (80.2%) patients in the oral information group (p = 0.84).

**Table 2:** Patient assessment of endoscopy procedure (gastroscopy or colonoscopy), Geneva and Lausanne, Switzerland, 2000.

	Written and oral information	Oral information	P value
Anxiety at time of procedure:			0.66
None	68 (24.5)	78 (26.1)	
Very slight	65 (23.4)	71 (23.7)	
Slight	62 (22.3)	64 (21.4)	
Moderate	50 (18.0)	51 (17.1)	
Strong	33 (11.9)	35 (11.7)	
How tolerable was procedure:			0.76
Very easy	59 (21.3)	68 (22.7)	
Quite easy	89 (32.1)	94 (31.4)	
Neither easy nor hard	63 (22.7)	69 (23.1)	
Quite hard	48 (17.3)	48 (16.1)	
Very hard	18 (6.5)	20 (6.7)	
Pain during procedure:			0.20
None	115 (41.4)	125 (41.8)	
Very slight	51 (18.3)	67 (22.4)	
Slight	26 (9.4)	38 (12.7)	
Moderate	58 (20.9)	45 (15.1)	
Strong	28 (10.1)	24 (8.0)	
Problems after procedure:			0.22
None	207 (75.3)	239 (80.2)	
Minor	61 (22.2)	52 (17.4)	
Moderate	6 (2.2)	6 (2.0)	
Severe	1 (0.4)	1 (0.3)	
Global assessment			0.82
Excellent	98 (36.2)	113 (38.2)	
Very good	92 (33.9)	98 (33.1)	
Good	64 (23.6)	62 (20.9)	
Fair	12 (4.4)	18 (6.1)	
Poor	5 (1.8)	5 (1.7)	

### Patient assessment of quality of information

Patients who received written information rated the overall quality of the information that they received higher than those who randomized to oral information only (Table 3). The differences were statistically significant for information about how to prepare for the procedure, the risks of the procedure, what to expect after the procedure, and for the global mean information score. In multivariate analysis, having received written information remained significantly associated with higher information quality scores (Table 4). In addition, men, younger patients, and hospital inpatients were also more satisfied with the information received than women, older patients and outpatients.

**Table 3:** Assessment of 8 quality-of-information items about endoscopic procedure in patients randomised to receive written leaflets or routine oral information.

	Prior information, mean* (SD) and median				
Information about:	Written and oral information		Oral information		P value †
	Mean (SD) and Median		Mean (SD) and Median		
Reasons for procedure	3.73 (1.21)	4	3.55 (1.34)	4	0.16
Alternative treatments or tests	2.23 (1.97)	3	2.02 (2.00)	2	0.25
How to prepare for the procedure	3.56 (1.34)	4	3.23 (1.60)	4	0.036
What will happen during the procedure	3.61 (1.23)	4	3.50 (1.40)	4	0.58
What the doctor will do during the procedure	3.75 (1.24)	4	3.60 (1.50)	4	0.70
Results and benefits you may expect from procedure	3.52 (1.38)	4	3.27 (1.58)	4	0.12
Possible risks and complications of procedure	3.24 (1.54)	4	2.26 (1.91)	2	<0.001
What you should expect after the procedure	2.99 (1.77)	3	2.59 (1.91)	3	0.020
Mean score	3.35 (1.06)	3.38	3.02 (1.22)	3.12	0.002

* score: 0: no information received, 1: poor, 2: fair, 3: good, 4: very good, 5: excellent.

† Mann-Whitney test

**Table 4:** Multivariate linear regression model predicting the patient global assessment of information received.

	Difference in mean information score	95% confidence interval	P value
Written information (vs oral)	0.32	0.12 to 0.51	0.002
Men (vs women)	0.22	0.02 to 0.41	0.029
Age 18–65 years (vs. 66–98 years)	0.44	0.24 to 0.65	<0.001
Inpatient (vs outpatient)	0.22	0.02 to 0.42	0.035

### Assessment of written materials

Of 278 respondents randomized to written information, 255 (92.1%) acknowledged receipt of a written document (appointment letter and written information), against 54 (18.1%) among the 299 respondents randomized to oral information (appointment letter only). Patients randomised to written information were further asked about their perception of these materials.

**Figure 2:**
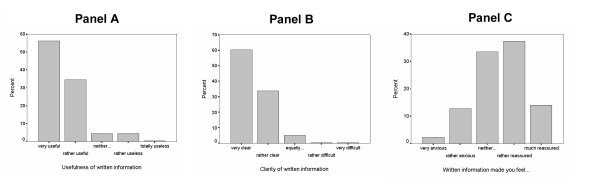
**(Panel A, Panel B, Panel C)**: Assessment of written information regarding digestive endoscopy by patients randomised to written information. Panel **A **illustrates whether information was useful or useless (N = 249). Panel **B **illustrates whether the written information was clear or difficult to understand (N = 252). Panel **C **illustrates whether the written information made the patient feel anxious or reassured (N = 257).

They rated the information as very useful (56.2%), rather useful (34.5%), neither useful nor useless (4.4%) rather useless (4.4%), totally useless (0.4%) (Figure 2, panel A). They also rated the information as very clear (60.3%), clear (33.7%), equally clear and difficult (5.2%), rather difficult (0.4%), very difficult (0.4%) (Figure 2, panel B). Finally they considered that the information has made them feel very anxious (2.3%), rather anxious (12.8%) neither anxious nor reassured (33.5%), rather reassured (37.4%), much reassured (14%) (Figure 2, panel C).

## Discussion

Informed consent is a legal and an ethical requirement, but there is a broad variety of legal prescriptions regarding informed consent in different countries. In Germany, for example, all aspects of the information (form content) are regulated in detail by legal authorities whereas in other European countries there is no legal requirement before a patient has access to endoscopy. The lack of such legal requirement has led us to conduct a randomised study aimed at evaluating the effects of combined written and oral information prior to endoscopy compared to oral information alone.

In the current study, of 912 patients primarily enrolled, 718 (78%) were eligible and only 577 completed all study procedures resulting in a dropout of about 46%. Although this rate is high, it reflects the real life of patients having to face with planned endoscopy.

Anxiety before endoscopy may have adverse consequences and can increase requirements for sedation and analgesics. Informing patients before gastrointestinal endoscopy may reduce the level of anxiety and therefore improve the patient tolerance during endoscopy. Moreover, obtaining informed consent from patients who will undergo a medical procedure is of particular interest for the doctor, since failure to obtain informed consent increases the likelihood of malpractice lawsuits. In our study, we found evidence that patients felt better informed if they had received an explanatory leaflet before their scheduled endoscopy procedure than if they were informed only verbally by their doctor.

Anxiety is sometimes put forward by physicians as a reason for withholding information from patients. Our results however do not support this attitude. Written information did not increase patient's level of anxiety prior to endoscopy. This was also suggested in previous studies [6,7]. A report in Norway even showed that patients were more interested in potentially alarming information such as complications than in technical aspects of the endoscopic procedures [8]. However, a document describing the risks of a procedure cannot be totally reassuring. In fact, we counted slightly more cancellations among patients in the written information group. Two possible explanations should be considered. The first is that some patients were made anxious by the written information, enough to lead them to cancel a beneficial procedure. The second is that the cancellations truly reflect informed decision making; some patients, when given the relevant information, decided that the risks were not worth the expected benefits. At the same time, patients in the written information group were much more satisfied with the information they received regarding risks. Globally, these results suggest that detailed information about an endoscopic procedure, including information about risks, is generally perceived as useful by patients, but that such information does not raise the levels of anxiety among patients who agree with the procedure.

Deficiencies in the structure of information spontaneously offered by physicians to their patients have been described previously. For instance, when physicians discuss routine clinical decisions with patients, they frequently describe the nature of their decisions but rarely alternatives, or risks and benefits of each option [9].

Even though the written leaflet was well accepted by most of our patients, a minority of them found that the written document was either useless or difficult to understand. Previous studies have shown that informed consent forms are often too complex for the average patient [10,11]. In one study evaluating consent forms used in radiology practice, the authors found that the most difficult forms to read were those written by hospital administrations [11]. They suggest that physicians should be involved in preparing these forms. Another study stressed the complexity and inadequacy of surgical forms [10]. The timing of the information is also important. A previous study showed that only 54% of patients had read a consent form given immediately before the endoscopy [12]. In contrast when the information sheet was sent two to four weeks before endoscopy 95% of patients had read it [12]. Shepherd et al [13] describe patient's satisfaction with information and consent forms sent by mail. These studies show that patients should be allowed to read information at home and discuss it with others. Agre et al evaluated alternative approaches in obtaining consent before colonoscopy or upper gastrointestinal endoscopy [14], and found that patients preferred videotapes, followed by doctor discussion. Luck et al also demonstrated the benefit of video information prior to colonoscopy [15] but Bytzer et al found that an information video shown to patients preparing for colonoscopy had no impact on tolerability or anxiety [16]. Hence several possibilities of improving the informed consent procedure can be explored and more studies are clearly needed to give a final proposal.

Our study has of course some limitations. The major one consists of an important dropout rate that reaches 46% of the primarily enrolled patients, a feature that can bias the results but also reflects the reel life in an open access referral center. The second is related to the content of the oral information given to the patient. Indeed, this content given by either the prescribing physician or/and by the gastroenterologist may vary to some extent (ie depending on the physician knowledge about endoscopy, personal fear and feeling), although the general content should be the same. This feature may also induce some bias in our study.

## Conclusion

In summary, our study found that structured and comprehensive written information is perceived as beneficial by patients, without negative impact on patient anxiety. Gastroenterologists should clearly explain to their patients the risks, benefits and alternatives of endoscopic procedures.

## Competing interests

The authors declare that they have no competing interests.

## Authors' contributions

CF, TP, JLF, conceived of the study. CF, TP performed the statistical analysis. CF, JLF, AH and TP drafted the manuscript. CR, IG, GD, AP participated in the design of the study. CF, JLF, GD participated in its coordination. All authors read and approved the final manuscript.

## Pre-publication history

The pre-publication history for this paper can be accessed here:


